# Prosthetic Corneal Surgery: A Narrative Review of Current Keratoprostheses and the Future Prospects of New Biomaterials

**DOI:** 10.3390/bioengineering13050548

**Published:** 2026-05-11

**Authors:** Łukasz Drzyzga, Dorota Śpiewak, Mariola Dorecka, Dorota Wyględowska-Promieńska

**Affiliations:** 1 Department of Ophthalmology, Prof. K. Gibiński University Clinical Center, Medical University of Silesia, 40-514 Katowice, Poland; 2 Clinical Ophthalmology Center Okolux, 40-754 Katowice, Poland; 3 Department of Ophthalmology, Faculty of Medical Sciences in Katowice, Medical University of Silesia, 40-287 Katowice, Poland

**Keywords:** corneal disease, corneal blindness, artificial cornea, corneal scaffolds, keratoprosthesis, osteo-odonto-keratoprosthesis, Boston keratoprosthesis, AlphaCor, EndoArt, CorNeat

## Abstract

Corneal diseases affect a significant proportion of the global population, with younger age groups being particularly vulnerable. In severe cases involving vision loss, standard techniques such as keratoplasty often fail. These cases require more invasive treatment through the implantation of artificial cornea or keratoprostheses. Since the development of the oldest technique for creating and implanting the first keratoprostheses by Strampelli, these devices have experienced major modifications to improve their biocompatibility, function, and long-term viability. As a result, there is currently a wide range of implants and procedures that are available and can be applied according to patient indications and clinical settings. This narrative review attempts to cover the literature on this topic by focusing on (1) recent advances in rigid (Boston Type I and II, osteo-odonto-keratoprosthesis, modified osteo-odonto-keratoprosthesis, and LV Prasad Eye Institute keratoprosthesis) and soft (AlphaCor, CorNeat, and EndoArt) keratoprosthesis designs and (2) more recent studies on innovative biomaterials and techniques that could lead to the fabrication of fully functional biosynthetic corneas with optimal properties. In our review of these materials, we cover the current clinical applications and limitations, as well as future prospects of less invasive and more efficient prostheses that could improve patient outcomes and quality of life.

## 1. Introduction

Corneal diseases are among the leading causes of blindness worldwide and affect populations in both developed and developing countries. It is estimated that approximately 10 million individuals are affected by bilateral corneal blindness globally. Notably, among children and young adults, corneal diseases are more frequent than any other ocular disease and are frequently associated with severe ocular surface damage [1,2]. The most common corneal conditions are keratoconus and keratitis: keratoconus is characterized by progressive thinning, protrusion, and irregular steepening of the cornea, and keratitis is characterized by inflammation of the cornea, which may be infectious or non-infectious in origin. The progression of keratoconus in young populations (10–25 years of age) is as high as 88% [3], with pediatric keratoconus showing a significantly higher progression rate than keratoconus in adult populations. Advanced stages may be associated with acute keratoconus, which may ultimately require corneal transplantation. Keratitis in children and young adults is often associated with trauma, contact lens wear, and allergic diseases, and constitutes a significant proportion of acute diseases in the young population. The main causes of keratitis are contact lens wear (36% of cases), followed by trauma (27% of cases), and eye diseases (12%). Keratoconjunctivitis, defined as inflammation involving both the cornea and the conjunctiva, predominantly affects children and young individuals and may lead to significant corneal complications. Overall, corneal diseases, such as keratoconus and keratitis, are more clinically significant and exhibit a more aggressive course and more rapid progression in children and young adults than in older adults.

Depending on the cause and extent of corneal disease, treatment strategies could involve topical and systemic medications, laser treatment, and surgical interventions, including corneal transplantation. The most common complication of corneal disease is corneal scarring, which occurs in approximately 71% of cases and may necessitate a lamellar or penetrating grafting procedure [4]. Importantly, vision loss due to corneal diseases is a significant clinical problem that can potentially lead to permanent visual impairment, especially with late diagnosis or rapid disease progression. In cases of vision loss from corneal damage, transplantation with tissue from deceased donors, known as keratoplasty, remains the treatment of choice. However, in cases of advanced disease involving severe scarring, neovascularization, severe dry eye, eyelid dysfunction, or repeated graft failures (even those preceded by limbal stem cell transplantation), conventional keratoplasty is often ineffective [5]. Under such circumstances, alternatives such as keratoprosthesis implantation become essential [6]. Some other common indications for keratoprosthesis implantation include chemical and thermal burns, as well as autoimmune diseases such as Stevens–Johnson syndrome, mucous membrane pemphigoid, Lyell’s syndrome, Sjögren’s syndrome, and autoimmune cicatrizing conjunctivitis. A keratoprosthesis is an artificial cornea designed to replace the damaged cornea and restore vision in patients with bilateral corneal blindness whose visual acuity is limited to light perception or hand-motion vision. The benefits of keratoprostheses vary according to clinical indications, ocular surface condition, and comorbidities; moreover, they offer only partial restoration of vision in cases of advanced ocular tissue damage, especially with concomitant retinal or optic nerve pathology. The implantation of these devices is also associated with limitations such as the risk of postoperative complications, the need for close ophthalmological monitoring, and limited long-term stability and functionality of the implant. Over the last few decades, there have been continuous advancements to overcome these limitations and improve clinical benefits for patients.

A fundamental requirement in the design of artificial corneas for use in humans is the ability to replicate the key characteristics of the native cornea. These characteristics include optical transparency, adequate mechanical strength, and high biocompatibility. Optical transparency defines the potential for visual rehabilitation; mechanical stability supports implant retention and structural durability; surface wettability modulates epithelial behavior and protein adsorption; and biocompatibility and resistance to microbial colonization are essential for minimizing chronic inflammation, infection, and device failure. Contemporary designs, including the Boston keratoprosthesis and the osteo-odonto-keratoprosthesis, have been developed to reduce long-term complications, such as corneal melt, chronic inflammation, infections and implant extrusion, through the incorporation of autologous or allogeneic tissues to facilitate implant biointegration [1]. There are several types of keratoprostheses available commercially, and the choice of device is based on disease etiology, clinical indications, ocular surface conditions, and tear film condition [5]. The long-term success of an artificial cornea is determined not only by surgical technique and device architecture, but also by the physicochemical and biological performance of the materials from which it is constructed. Thus, the design of keratoprostheses and the evolution of biomaterials should be viewed not as separate disciplines, but as interdependent pillars of a unified therapeutic strategy for severe corneal blindness. Accordingly, each generation of keratoprostheses reflects the progress of biomaterials science—from early rigid polymer-based devices, through hydrogel interfaces and nanofibrous scaffolds, to next-generation regenerative platforms engineered for active integration with host tissues. As these fields continue to converge, the distinction between prosthetic replacement and bioengineered corneal regeneration is expected to become increasingly blurred.

The current literature on the bioengineering of corneal implants is vast and challenging to navigate. While there have been previous reviews on the topic, they have primarily focused on single devices, specific surgical techniques, or the historical evolution of keratoprostheses. The present narrative review tries to fill in this gap by providing an integrated, multidimensional, and up-to-date overview of the entire field of prosthetic corneal surgery. The unique features of this review are that (1) it covers the major aspects of existing devices as well as newer technologies and their future clinical prospects; (2) it integrates and presents clinical and bioengineering perspectives by discussing both surgical aspects and modern biomaterials and technologies; (3) it presents an analytical and comparative approach by including both qualitative and quantitative data on the major keratoprosthesis platforms, as well as a critical evaluation of their strengths, limitations, and current clinical maturity; (4) it presents the most recent advances by including publications from 2023 to 2026, particularly in rapidly evolving areas such as corneal regeneration and artificial intelligence applications in ophthalmology; and (5) it presents a future-oriented perspective by addressing translational readiness, regulatory challenges, and manufacturing scalability. The first part of the review brings together the current available knowledge on keratoprostheses in clinical use, their features and functions, and complications and limitations. The second part of this review is dedicated to ongoing and upcoming research on novel biomaterials and technologies that could improve the current features and functions of keratoprostheses. We believe that the collated information presented here will be a useful resource for both clinicians and researchers.

## 2. Materials and Methods

### 2.1. Study Design

This manuscript was prepared as a narrative review to provide a comprehensive overview of currently available keratoprosthesis devices and their clinical indications, surgical techniques, postoperative complications, and future directions in corneal biomaterials, tissue engineering, and regenerative medicine.

### 2.2. Search Strategy

A literature search was conducted using the electronic databases PubMed, Scopus, and Web of Science. Studies published up to January 2026 were considered.

The search strategy included combinations of the following keywords: keratoprosthesis, artificial cornea, Boston keratoprosthesis, Boston keratoprothesis, osteo-odonto-keratoprosthesis, Modified Osteo-Odonto-Keratoprosthesis, CorNeat, AlphaCor, EndoArt, corneal biomaterials, corneal scaffold, corneal regeneration, 3D bioprinting, and tissue engineering.

Additional relevant publications were identified through manual screening of the reference lists of selected articles.

### 2.3. Eligibility Criteria

Based on the eligibility criteria, we included (1) peer-reviewed articles published in English, (2) original clinical studies, (3) experimental or translational studies, (4) review articles, (5) case series or clinically relevant case reports, (6) landmark publications of historical relevance, and (7) studies related to keratoprostheses, artificial corneas, corneal substitutes, biomaterials, or regenerative strategies. The following publications were excluded: (1) duplicate records, (2) conference abstracts without full-text availability, (3) non-peer-reviewed materials, (4) studies outside the scope of the review, and (5) publications with insufficient scientific or clinical relevance.

### 2.4. Study Selection and Data Synthesis

Titles and abstracts were screened for relevance to the objectives of this review. Full texts of potentially eligible studies were subsequently assessed. The selected literature was narratively synthesized and organized into thematic sections addressing keratoprosthesis types, indications, outcomes, complications, biomaterials, and future perspectives.

### 2.5. Methodological Considerations

As this work was designed as a narrative review, standard systematic review tools such as PRISMA flowcharts, risk-of-bias assessment, and meta-analytic statistical methods were not applied.

## 3. Types of Keratoprostheses

### 3.1. Rigid Keratoprostheses

Rigid keratoprostheses are primarily manufactured from polymethyl methacrylate (PMMA), a rigid polymer, and are equipped with protective components that provide mechanical stability and resistance to intraocular pressure and ocular movements. This group of keratoprostheses include the Boston keratoprostheses, the osteo-odonto-keratoprosthesis (OOKP), and its modified version, the modified osteo-odonto-keratoprosthesis (MOOKP) [1].

#### 3.1.1. Osteo-Odonto-Keratoprosthesis and Modified Osteo-Odonto-Keratoprosthesis

**Figure 1:**
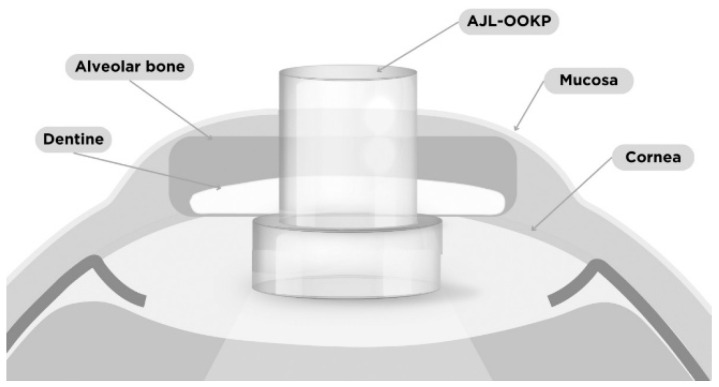
Components and Structure of Osteo-Odonto-Keratoprosthesis [7]. The figure was adapted with permission from AJL Ophthalmic 6 May 2026.

Among the various available techniques for keratoprosthesis implantation, one of the oldest and most widely used, since the 1960s, is the Strampelli technique or OOKP (Figure 1). Synthetic implants frequently fail due to a fibrovascular reaction in the host tissue that leads to implant resorption or extrusion. Instead, OOKP utilizes fully autologous tissue to enable stable, long-term integration with host tissues [5]. Commonly referred to as “tooth-in-eye” surgery, this is an innovative procedure used in cases of severe corneal blindness when conventional corneal transplantation is not feasible. The surgical procedure involves using (1) the patient’s own tooth to provide structural support, (2) the alveolar bone for stability, and (3) the buccal tissues to supply vascular support—all of which support an optical cylinder made of PMMA [1,5] and ensure both protection and nourishment for the keratoprosthesis. The OOKP has undergone numerous modifications since its inception, and the surgical technique has been repeatedly refined. In particular, this prosthesis is associated with the relatively frequent occurrence of laminar bone resorption, and the approaches to overcome this limitation involve the use of an autoclavable μ-milling device, bone morphogenetic proteins, and bisphosphonate therapy [1].

**Figure 2:**
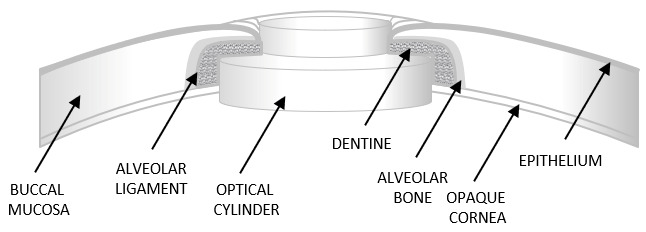
Components and Structure of the Modified Osteo-Odonto-Keratoprosthesis [9]. Created by the authors based on material from Ento Key.

In the mid-1990s, Falcinelli refined Strampelli’s technique by introducing several modifications, including a larger biconvex optical cylinder, cryoextraction of the crystalline lens, and the use of intact buccal mucosa [5,8]. These improvements form the basis of the contemporary MOOKP (Figure 2), which is preferred in severe ocular surface disease. This type of keratoprosthesis (MOOKP) is used in conditions such as Stevens–Johnson syndrome, Lyell’s syndrome, Sjögren’s syndrome, mucous membrane pemphigoid, severe chemical ocular injuries, advanced keratinization of the ocular surface, severe ocular infections, acid and other chemical burns, radiation-induced ocular damage, and bullous keratopathy secondary to glaucoma surgery, as well as in cases of failed corneal transplantation [6].

The procedure for OOKP and MOOKP involves a multistage intervention usually performed over several months [6,10,11,12]. In the first stage, the ocular surface is prepared with buccal mucosal grafting, and an osteo-odonto-acrylic lamina is fashioned from the patient’s tooth–bone complex to support the optical cylinder. The lamina is temporarily implanted in a vascularized subcutaneous pocket to allow biological maturation [6,13]. In the second stage, the matured lamina is implanted onto the eye and the optical cylinder is aligned with the visual axis to restore retinal light transmission [5,6]. In selected patients lacking suitable teeth, alternative biological support with tibial bone or donor tooth grafts have been described, although long-term outcomes may be less favorable because of progressive resorption [14,15]. In a small group of patients in whom OOKP implantation cannot be performed due to the absence of suitable teeth and healthy eyelid skin, another model has been developed—the Type II “Lux” keratoprosthesis. Structurally, it is similar to the MOOKP and consists of a PMMA optical lens, a titanium posterior plate, and a titanium sleeve. However, this technique also requires corneal transplantation [1,16]. Despite its complexity, this multistage approach remains one of the most durable solutions for end-stage ocular surface disease.

After the procedure, topical broad-spectrum antibiotics need to be applied every night for the duration of the patient’s life. Maintenance of lamina stability requires a balance between bone resorption and bone remodeling processes. The osteo-odonto-acrylic lamina is prone to excessive resorption, a process that usually advances slowly and often goes undetected due to its position beneath the oral mucosal graft. Laminar resorption may lead to thinning of the lamina and, consequently, to decentration of the optical cylinder, refractive disturbances, keratoprosthesis leakage, ocular hypotony, and endophthalmitis [13]. Laminar resorption represents a late and progressive complication of MOOKP, most frequently observed within 2–5 years following implantation, although earlier onset has been reported [6].

MOOKP necessitates a multidisciplinary approach involving ophthalmic surgeons, maxillofacial surgeons, and, in some cases, orthopedic specialists. It is based on keratoprostheses incorporating autologous biological haptics, such as the OOKP and tibia keratoprosthesis [17]. Careful patient selection, meticulous surgical technique, and long-term follow-up are essential for achieving favorable outcomes [5]. In addition, MOOKP is associated with several potential clinical challenges, including maxillofacial, mucosal, and prosthesis-related complications that may occur at different stages of treatment. However, this method demonstrates high biointegration and favorable long-term anatomical and functional outcomes resulting from durable integration of the implant with the patient’s autologous tissues and the maintenance of visual function despite complex ocular surface reconstruction. For these reasons, despite its complexity and limited indications, it remains an important therapeutic option, particularly for patients with bilateral corneal blindness and severe ocular surface disease, and is among the last-resort therapeutic options for end-stage corneal blindness [1,5,18,19].

#### 3.1.2. Boston Type I Keratoprosthesis

**Figure 3:**
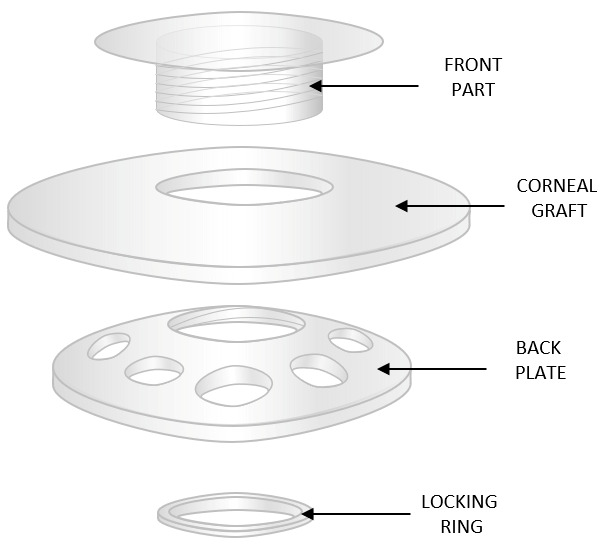
Assembly of the Boston Type I keratoprosthesis (Image courtesy of EyeWorld.org). Created by the authors based on an illustration from EyeWorld.org, courtesy of EyeWorld.org.

The Boston Type I keratoprosthesis (Figure 3) (Massachusetts Eye and Ear, Boston, MA, USA), consists of a front plate composed of PMMA, with a central diameter of 3.5–3.7 mm, and a back plate composed of either PMMA or titanium. A titanium locking ring is attached to the posterior plate. Initially, the back plate was designed as a solid PMMA disc with a diameter of 8 mm, but this feature limited nutrient diffusion and predisposed patients to keratolysis, i.e., thinning of the peripheral corneal stroma and the development of epithelial defects from autoimmune-mediated inflammatory processes. Currently, the back plate has a diameter of 8.5 mm and incorporates 16 fenestrations that facilitate nutrient diffusion, thereby reducing the incidence of keratolysis from 50% to 10% In contrast to the use of dental and buccal tissue in OOKP or MOOKP, the Boston Type I keratoprosthesis utilizes a donor corneal graft as a carrier positioned between the anterior and posterior plates of the device [20,21]. Comparative studies have demonstrated that both fresh and frozen donor corneas provide similar safety and efficacy as carrier grafts, with comparable visual outcomes and no increased incidence of complications such as corneal melt, leakage, or endophthalmitis [22,23]. The procedure largely overlaps with the general principles of keratoprosthesis implantation described above, including preparation of the ocular surface, removal of the diseased corneal tissue, and placement of the prosthetic device. However, in contrast to OOKP, the Boston Type I keratoprosthesis does not require harvesting of autologous dental or buccal tissue. Instead, the device is assembled with a donor corneal graft acting as a carrier, through which the optical stem is inserted, and the entire complex is then sutured into the host cornea using techniques similar to penetrating keratoplasty. This approach makes the procedure less invasive and technically less complex, although it remains dependent on adequate ocular surface conditions.

Earlier generations of Boston keratoprostheses were primarily based on a carrier graft designed to anchor the implant within the patient’s cornea, achieving only limited and often temporary integration [20,24]. Moreover, standard Boston keratoprostheses require donor corneal tissue, which remains limited in availability [20,25]. These procedures involve major surgical intervention, often require multiple stages, and may be associated with procedure-specific complications. Over the years, several modifications have been introduced to the Boston keratoprosthesis. For example, the addition of holes in the back plate has improved nutrient diffusion and significantly reduced the incidence of keratolysis. Another approach involves replacing the PMMA with a titanium back plate that snaps onto the stem without a locking ring and provides a streamlined assembly. Similarly, PMMA back plates with a threadless, snap-on design are also used, with the aim of reducing corneal graft damage associated with component screwing during surgery and decreasing the risk of retroprosthetic membrane (RPM) formation from fibrovascular tissue proliferation behind the implant [26,27]. A threadless stem also has the advantages of easier handling and greater cost efficiency, as it is manufactured by molding instead of machining processes [20]. Titanium is well tolerated by surrounding tissues, exhibits high corrosion resistance, and is both lightweight and durable. The use of a titanium coating can enhance the adhesion of PMMA to corneal tissue. Furthermore, as it is non-magnetic, patients with such implants can safely undergo magnetic resonance imaging. Finally, the titanium back plate can be anodized to achieve a blue or brown color, enhancing the device’s aesthetic appearance [28]. However, both titanium and PMMA could promote the formation of an RPM, which may lead to decreased visual acuity, obstruction of the visual axis, and, in some cases, secondary complications such as glaucoma or inflammation, potentially necessitating additional surgical interventions and contributing to functional failure of the implant [29].

Indications for the use of the Type I Boston keratoprosthesis are classified by prognosis, i.e., the anticipated clinical outcome and risk of adverse events. A favorable prognosis is usually assumed in patients undergoing penetrating keratoplasty or pars plana vitrectomy with silicone oil tamponade, those with a history of keratitis (particularly viral), and individuals with congenital aniridia. In contrast, unfavorable outcomes are expected in immune-mediated conditions such as Stevens–Johnson syndrome and ocular cicatricial pemphigoid, as well as in severe chemical burns accompanied by eyelid deformities [10,11]. It has also been shown that patients with severe ocular surface disease benefit from optimization of the ocular surface before keratoprosthesis implantation, e.g., through limbal stem cell transplantation, as this improves surgical outcomes [1].

#### 3.1.3. Boston Type II Keratoprosthesis

**Figure 4:**
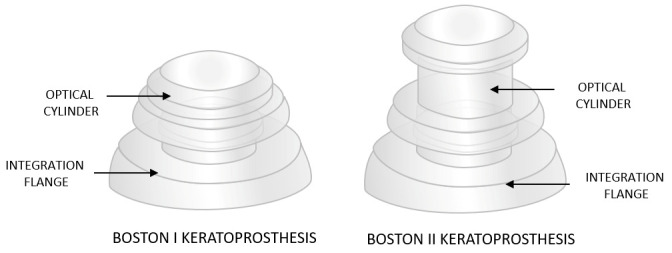
Boston Keratoprosthesis Design: Type I (**left**) and Type II (**right**). Created by the authors based on F. Yaghouti et al. [30].

The Boston Type I and II keratoprostheses (Massachusetts Eye and Ear, Boston, MA, USA), share a similar design. The key features of Type II that distinguish it from Type I are its elongated anterior stem and the implantation of the optic component through surgically closed eyelids (Figure 4). This is achieved by creating an opening in the upper eyelid for the optic component to emerge.

Type II Boston keratoprosthesis is used less frequently than Type I and is intended for patients with the most severe forms of cicatrizing ocular surface diseases, such as Stevens–Johnson syndrome and mucous membrane pemphigoid, and patients with severe eyelid dysfunction and end-stage dry eye disease. Boston Type II keratoprosthesis is implanted in only a limited number of specialized centers, as it is considered a “last-resort” procedure reserved for the most advanced cases of corneal disease. It requires a high level of surgical expertise and interdisciplinary care and is associated with a substantial risk of complications. The technique necessitates permanent tarsorrhaphy, through which the anterior portion of the prosthesis (the so-called nub) is exteriorized. This procedure is technically more demanding than Boston Type I implantation and is frequently associated with serious complications, including glaucoma (potentially vision-threatening), RPM formation, and implant extrusion. Patients undergoing Boston Type II keratoprosthesis implantation require strict, long-term follow-up, often including systemic immunosuppression and coordinated care involving multiple specialists [31,32].

#### 3.1.4. LV Prasad Eye Institute Keratoprosthesis

A modified version of the Boston Type I keratoprosthesis, LV Prasad Eye Institute keratoprosthesis (LVPKP) (Hyderabad, India), is an innovative artificial corneal implant designed at the LV Prasad Eye Institute. This keratoprosthesis is indicated for severe, bilateral corneal diseases associated with complete absence of the tear film (xerophthalmia) and ocular surface keratinization, in patients who have sustained chemical or thermal burns, and in conditions such as Stevens–Johnson syndrome. It was specifically developed to address end-stage corneal blindness in patients suffering from severe dry eye disease, including those with Stevens–Johnson syndrome, for whom conventional corneal transplantation is either ineffective or contraindicated. LVPKP is considered an effective alternative to more invasive procedures, such as the MOOKP, especially in cases where those procedures are contraindicated.

The LVPKP features an elongated optical stem, approximately 0.75 mm longer than that of the standard Boston device. This design allows keratoprosthesis implantation beneath a labial mucosal graft while preventing excessive mucosal overgrowth onto the optical portion of the device [33,34]. The procedure is performed in two stages. In the first stage, a labial mucosal graft is harvested and placed over the deepithelialized ocular surface. After approximately three months, in the second stage, the keratoprosthesis is implanted.

The advantages of this technique include more rapid improvement in visual function compared with other approaches, a retention rate exceeding 80% at 3–5 years, and a simpler surgical procedure compared with the MOOKP. Furthermore, this technique does not require the use of the patient’s tooth, as is necessary in MOOKP, and allows for the administration of topical ophthalmic medications, unlike the Boston Type II keratoprosthesis. The most common complications include the formation of an RPM (observed in approximately 43% of cases) and glaucoma (observed in approximately 26% of patients) [33].

### 3.2. Soft Keratoprostheses

To enhance the biointegration of keratoprostheses, skirts composed of soft materials based on various synthetic polymers have been introduced. Such keratoprostheses are referred to as soft keratoprostheses. The AlphaCor keratoprosthesis was the first soft keratoprosthesis to receive FDA approval approximately two decades ago. Since then, other soft synthetic keratoprostheses, such as CorNeat and EndoArt [1], have also been developed.

#### 3.2.1. AlphaCor Keratoprosthesis

**Figure 5:**
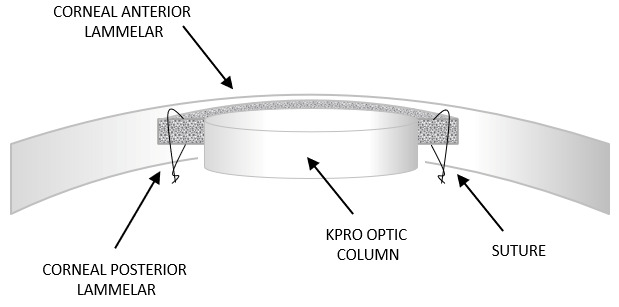
Schematic Representation of the AlphaCor Keratoprosthesis Implanted Intrastromally with the Optic Column [36]. Created by the authors based on Xiang J. at al.

**Figure 6:**
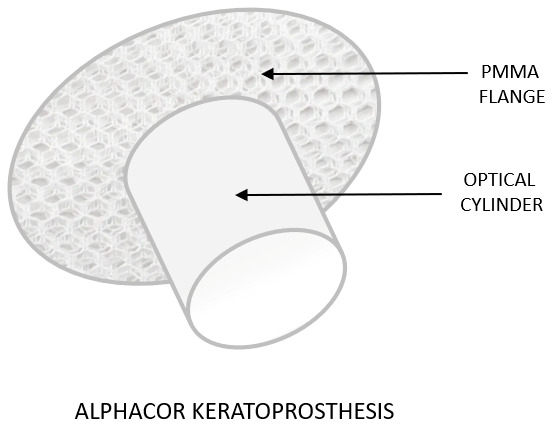
AlphaCor Keratoprostheses [36]. Created by the authors based on Xiang J. at al.

AlphaCor (Coopervision Surgical Inc., Lake Forest, CA, USA) is a synthetic cornea composed of poly(2-hydroxyethyl methacrylate) (PHEMA), which is a one-piece, hydrophilic construct produced by polymerization. The device consists of a biointegrable, sponge-like skirt surrounding the optic component, which is a transparent lens with high refractive power. Both the central and peripheral components are chemically identical; however, they differ in water content. The skirt contains a higher water fraction, which gives it greater porosity and thereby facilitates biointegration with the surrounding tissue. The two concentric regions, i.e., the skirt and the optic center, are joined at the interface by an interpenetrating polymer network. The peripheral portion of the keratoprosthesis contains interconnecting pores, which enable integration of the prosthetic material with the surrounding host corneal tissue [35]. The device has an overall diameter of 7.0 mm, a thickness of 0.6 mm, and a surface curvature designed to provide appropriate refractive power following implantation into the recipient cornea. The keratoprosthesis is available in two variants depending on the optical power of the central component: AlphaCor-A (for aphakic patients) and AlphaCor-P (for phakic or pseudophakic patients). A schematic illustration of the intrastromal keratoprosthesis is shown in Figure 5, while an image of the AlphaCor keratoprosthesis is presented in Figure 6. These clearly demonstrate the transparent optical zone and the sponge-like skirt; the transition between the two components is smooth and lacks a distinct boundary [36].

The implant is placed within a surgically created lamellar corneal pocket. Following placement of the implant within the corneal pocket, the procedure proceeds in two distinct stages: the tissue posterior to the optical component is removed during the first stage, whereas the tissue anterior to the optical component is excised during the second stage. The porous skirt remains enclosed within the corneal stroma, where it undergoes biointegration through cellular colonization and collagen deposition [37].

One of the limitations of the AlphaCor prosthesis is the high water content in the skirt that results in large pores. The large size of the pores may lead to insufficient suture retention and reduced mechanical strength of the implant. This may result in stromal softening of the cornea to which the keratoprosthesis is affixed and, eventually, implant extrusion, which is a serious complication. This limitation is overcome in a modified version of AlphaCor that is in the form of a T-shaped keratoprostheses (also composed on PHEMA hydrogel) in which hyaluronic acid and cationized gelatin is incorporated into the skirt to enhance cell adhesion and promote stronger integration of the implant with host tissue. In addition, polyethylene glycol (PEG, a biocompatible polymer) is applied in the inferior portion of the optic column to reduce the risk of RPM formation by limiting cell adhesion and proliferation [36].

#### 3.2.2. CorNeat Keratoprosthesis

The CorNeat keratoprosthesis (CorNeat Vision Ltd., Ra’anana, Israel) is a fully synthetic corneal implant designed for the treatment of advanced corneal blindness in eyes unsuitable for conventional keratoplasty. The device is composed of a central optical element and an external integrating skirt manufactured from electrospun carbonated polyurethane nanofibers. Its design is based on extra-corneal biointegration, in which the implant is anchored beneath the conjunctiva rather than relying exclusively on corneal stromal fixation. This keratoprosthesis does not require donor corneal tissue or support from other surgical specialties, making it an attractive alternative. Its innovative concept is based on subconjunctival integration and a microporous matrix designed to promote cellular ingrowth. That is, its structure enables biointegration between the synthetic optical component and the surrounding ocular tissues. This concept represents a shift from classical intrastromal carrier-based keratoprostheses toward a subconjunctival integration model that takes advantage of the rich vascularity and fibroblast population of Tenon’s capsule and conjunctival tissues. Migration of host fibroblasts into the porous skirt is intended to create stable long-term anchorage while reducing the risk of corneal melt, stromal necrosis, or inadequate graft support that may occur with donor-dependent systems [38]. The CorNeat keratoprosthesis is intended for both phakic and pseudophakic patients, and there are plans for the future development of devices for aphakic patients.

**Figure 7:**
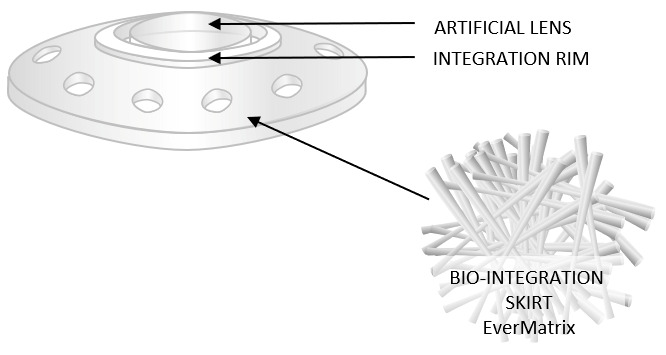
Components and Structure of the CorNeat Keratoprosthesis [39]. Created by the authors based on Litvin G. at al.

The skirt component of CorNeat is approximately 250 µm thick and extends about 5 mm from the optical zone edge. It terminates about 1 mm from the nearest insertion of the extraocular muscles. Its dimensions fit most eyes and allow easy trimming and intraoperative customization (Figure 7). The optical component is made of PMMA and provides a fixed optical power of 40.8 diopters, approximately equal to the natural cornea’s optical power of 42 diopters. The surfaces of the CorNeat optic component are spherical; this helps minimize the risk of astigmatism resulting from implant malposition or decentration. Furthermore, the optical component has a 10 mm diameter, which provides an effective optical zone of approximately 6.5 mm and potentially enables a physiologically normal field of vision. In addition, the optical component features a posterior corneal groove on its back surface that conforms to the patient’s corneal edge. The implant also has three pairs of apertures, spaced at 120° intervals, for surgical suture placement. In addition, a series of integration openings filled with nanofibers promotes biointegration with host tissues. Four ports are placed circumferentially around the optical element to provide access to the anterior chamber. These ports allow for future surgical interventions, such as the implantation of a foldable intraocular lens. One port, with a width of 2.6 mm, is specifically optimized for this purpose.

To facilitate implantation of the CorNeat, a set of positioning markers is incorporated along the edge of the optical zone. The outer component of the implant, i.e., the skirt, is placed beneath the conjunctiva, a highly vascularized tissue rich in fibroblasts. This environment encourages fibroblasts from Tenon’s capsule to migrate into the skirt, potentially improving the implant’s biointegration with ocular tissues [33,34]. After implantation, the CorNeat resembles a natural cornea and provides a favorable cosmetic outcome [38].

One of the advantages of CorNeat is that the surgical workflow is less complex than that for other keratoprostheses such as MOOKP: it involves fitting the implant into the patient’s trephined cornea using dedicated instruments and securing it to the eye with only three sutures. In addition, the operative time is approximately 45 min, which is significantly shorter than that required for other keratoprosthetic procedures. Other advantages include independence from donor tissue requirement, integration of synthetic optical components with ocular tissue via subconjunctival implantation, an optimized visual field due to a wide optical zone, and favorable cosmetic outcomes [38]. In addition, because fixation is primarily extra-corneal, the platform may theoretically reduce some complications related to stromal carrier degradation.

Preclinical rabbit implantation studies and early human experience have demonstrated the feasibility of this approach, with retention of the implant and restoration of a transparent optical axis in selected cases. Histopathological analyses have shown fibroblast colonization and collagen deposition within the porous skirt and biosealing openings, supporting active tissue integration. A localized foreign-body response involving macrophages, multinucleated giant cells, and lymphocytes has also been observed, predominantly at the implant periphery, suggesting controlled host remodeling rather than diffuse inflammatory failure [38,40]. However, clinical evidence is still limited to early case reports and small series, and long-term data regarding retention, glaucoma progression, infection risk, conjunctival retraction, endothelial safety, and visual durability are not yet sufficient. As with all emerging keratoprosthesis technologies, broader adoption will depend on multicenter validation, reproducible surgical outcomes, and long-term post-market surveillance. Nevertheless, CorNeat represents one of the most innovative fully synthetic approaches in contemporary corneal prosthetic surgery and may become an important donor-independent option in the future.

#### 3.2.3. EndoArt Endothelial Keratoprosthesis

**Figure 8:**
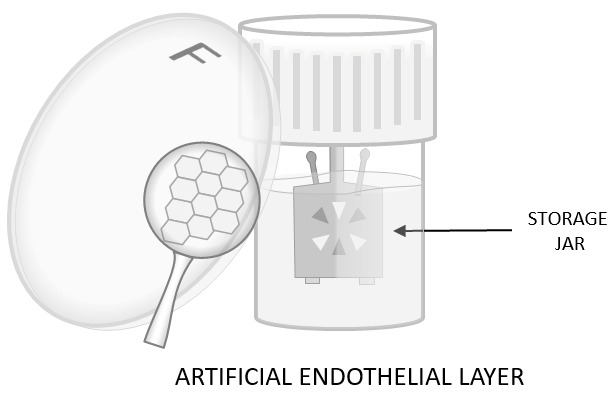
EndoArt Endothelial Keratoprosthesis. Schematic illustration of the artificial endothelial layer and storage jar (Created by the authors based on material from the website https://eye-yon.com/endoart/, Accessed on 22 April 2025).

EndoArt represents the first synthetic implant designed to replace the human corneal endothelium, which—unlike many other tissues—lacks regenerative capacity. This unique device enables the treatment of chronic corneal edema associated with endothelial failure using a minimally invasive surgical technique, while eliminating the need for donor tissue. The EndoArt endothelial keratoprosthesis (EyeYon Medical Ltd, Ness Ziona, Israel) (Figure 8) is a thin, flexible artificial endothelial layer made of a biocompatible, hydrophilic acrylic copolymer. The implant has a dome-shaped posterior lamellar component that conforms to the posterior corneal curvature. Acting as an inert physical barrier, this lamellar component prevents the aqueous humor from entering the posterior corneal stroma. EndoArt consists of an optically transparent disc with a diameter of approximately 6–8 mm; the radius of curvature is individually tailored to match the posterior corneal surface of the patient, typically 6–7 mm. The thickness ranges between 30 and 50 μm. The optically transparent plate has a diameter and radius of curvature of 6.0–8.0 mm and a thickness of 30–50 μm [41,42].

The surgical technique for implanting the EndoArt closely resembles that for Descemet membrane endothelial keratoplasty [43]. The procedure starts with corneal epithelial debridement to enhance visualization and the creation of three limbal ports, following which a descemetorhexis with a diameter of approximately 7.0–7.5 mm is performed. An excessively large descemetorhexis may result in implant laxity, whereas an undersized descemetorhexis may lead to implant detachment or inversion. After completion of the descemetorhexis, the EndoArt keratoprosthesis is positioned on the posterior corneal surface, specifically on the posterior stroma. The device is assembled or loaded and inserted into the anterior chamber through the incision, then unfolded and centered on the posterior stroma [43]. Next, to achieve proper apposition of the implant to the cornea, gas, most commonly perfluoropropane (C_3_F_8_), is injected into the anterior chamber. The gas should fill approximately 80% of the anterior chamber volume, without inducing pupillary block. The implant is further stabilized using one to three transcorneal fixation sutures, with one suture typically placed at the 12 o’clock position. External massage of the corneal surface is then performed to eliminate air bubbles that may accumulate between the implant and the corneal stroma [44].

Because the EndoArt implant is synthetic and acellular, it is more durable and less prone to manipulation, without the risk of endothelial cell loss or graft rupture that is associated with donor tissue. This durability may reduce intraoperative complications [43]. This implant may be particularly beneficial in patients who experience graft rejection after Descemet membrane endothelial keratoplasty or Descemet stripping automated endothelial keratoplasty. Patients at high risk of graft failure may also benefit from this approach, including those with a history of multiple glaucoma surgeries, prior lamellar keratoplasty, recurrent uveitis, and structural anterior segment abnormalities such as aniridia and anterior synechiae [41,44]. The use of EndoArt may result in significant improvement in visual function and a reduction in corneal thickness, even in patients with advanced corneal edema. This effect is attributed to the limited penetration of aqueous humor into the corneal stroma and the consequent reduction in stromal edema [41]. The preliminary clinical outcomes with this artificial endothelial layer substitute are promising, with encouraging postoperative outcomes reported to date. For example, several studies have demonstrated significant reductions in corneal thickness; markedly edematous corneas measuring more than 800 µm preoperatively decreased to approximately 550–600 µm following implantation. EndoArt implantation also eliminates the need for donor tissue, thereby avoiding the risk of immune rejection and reducing the requirement for long-term corticosteroid therapy [43]. According to the available literature, 82% of treated patients had previously undergone at least one corneal transplant, while nearly 40% had undergone three or more procedures. In addition, concomitant ocular comorbidities, including previous glaucoma surgery, were present in 78% of patients [43,45,46]. However, it should be noted that the EndoArt procedure is still under development, and efforts are ongoing to improve its efficacy and safety.

## 4. Comparative Analysis of Current Keratoprosthesis Platforms

The currently available keratoprosthesis systems differ substantially in their design philosophy, biomaterials, surgical complexity, degree of biological integration, and target patient populations. While each platform has specific advantages and limitations, no single device is universally optimal for all forms of corneal blindness. Therefore, a comparative evaluation is essential to better understand their current clinical roles, unmet needs, and future potential. Because currently available keratoprosthesis systems differ not only in design and surgical philosophy, but also in clinical performance and evidence maturity, both qualitative and quantitative comparison are required. However, published quantitative outcomes, where available, should be interpreted cautiously, as direct comparisons are limited by heterogeneity in indications, surgical expertise, follow-up durations, and study designs. Table 1 summarizes the principal structural and clinical characteristics of the major platforms, and Table 2 presents selected outcome measures and the current level of clinical evidence.

As depicted in these tables, currently available keratoprosthesis systems differ substantially in their design philosophy, surgical requirements, target indications, biological integration, and long-term outcomes.

Boston keratoprosthesis and OOKP/MOOKP currently represent the most established platforms, supported by the strongest long-term clinical evidence. Particularly, OOKP/MOOKP remains the most robust option for the most advanced cicatrizing ocular surface disorders, particularly in eyes with absent tear production, keratinization, and severe adnexal disease. Its principal advantage is durable long-term biointegration achieved through autologous tissues. However, this benefit comes at the cost of extreme surgical complexity, multistage reconstruction, and limited worldwide availability. Boston keratoprosthesis remains the most widely adopted option for severe corneal blindness in eyes with partially preserved ocular surface function. It offers broad clinical experience and favorable visual rehabilitation in appropriately selected patients, but requires lifelong surveillance because of glaucoma, RPM formation, and other device-related complications. In contrast, CorNeat and EndoArt are newer technologies with promising donor-independent concepts, but with more limited follow-up and smaller published cohorts.

CorNeat represents a next-generation fully synthetic design intended to reduce dependence on donor tissue and simplify implantation. Its biomimetic subconjunctival integration strategy is particularly attractive from a translational perspective. Nevertheless, there are limitations posed by lack of concrete data on long-term retention, complication rates, and reproducibility, and multicenter validation studies are required.

EndoArt differs fundamentally from the other systems because it is designed not for total corneal blindness caused by ocular surface failure, but for endothelial dysfunction and chronic corneal edema. It may address an important unmet need in patients at high risk of endothelial graft failure, although long-term durability and optimal selection criteria remain under investigation.

Overall, no single keratoprosthesis is ideal for all clinical scenarios. While these numerical data should be interpreted with caution because of the heterogeneity in the literature, they illustrate the relative maturity of established devices and the emerging potential of newer platforms. Importantly, these devices should not be viewed as direct competitors, but rather as complementary solutions for distinct forms of severe corneal blindness. That is, the four platforms represent distinct therapeutic principles rather than directly interchangeable solutions. Device selection should, therefore, be individualized according to ocular surface status, tear film function, eyelid anatomy, glaucoma risk, posterior segment potential, and access to long-term specialist follow-up.

## 5. Complications of Keratoprostheses

Although keratoprosthesis technologies may provide meaningful visual rehabilitation in eyes with otherwise poor prognosis, all currently available devices remain associated with significant short- and long-term complications. While graft failure following corneal transplantation is relatively less frequent, complications associated with keratoprostheses are frequent and may also occur in the late postoperative period [30,47]. Importantly, the type and frequency of adverse events vary according to implant design, ocular surface status, patient selection, and duration of follow-up. A detailed overview of the major complications associated with these procedures is discussed in the following section.

Complications associated with OOKP may occur at various stages of surgical management—from the preparatory phase, through the early postoperative period, to long-term follow-up. Complications arising in the pre-implantation stage are a serious concern and include damage to teeth and oral structures, such as the formation of oroantral fistulae (approximately 6%) and mandibular fractures (rare, usually <1–2%); trophic changes in the oral mucosa, such as necrosis, ulceration, and infection (have been reported in 8–46%), as well as the development of mucosal cysts (may occur in 5–10%) and hematomas [48,49,50]; and scarring at the site of buccal mucosal graft harvesting [51]. These complications can be managed with appropriate surgical intervention, targeted antimicrobial therapy, and meticulous postoperative care, although they may increase procedural complexity and overall morbidity. Their consequences may include delayed healing, structural instability of the implant, increased risk of infection, and, in severe cases, failure of subsequent surgical stages [5,52].

MOOKP implantation is associated with serious postoperative complications, including elevated intraocular pressure, the development of secondary glaucoma, and progression of pre-existing glaucoma (in approximately 35–65%) [20,48,53,54]. Monitoring for glaucoma in patients with a keratoprosthesis primarily involves visual field assessment and evaluation of the optic nerve head. Glaucoma drainage devices, particularly the Ahmed glaucoma valve, are considered especially effective in the management of glaucoma in patients with the OOKP implantation. Furthermore, implantation of OOKP prior to buccal mucosal grafting stabilizes IOP in approximately three-quarters of patients with coexisting glaucoma [55,56].

The most common significant long-term complications of OOKP/MOOKP involve complex interactions between the integrity of the mucosal covering and posterior segment structures, such as the vitreous body and retina, as well as the stability of the prosthesis itself. These complications include retinal detachment (3–26%), choroidal detachment (1–8%), endophthalmitis (2–8%), vitreous hemorrhage (2–8%), formation of epiretinal membranes (5–15%), cystoid macular edema (4–12%), and retinal pigment epithelium atrophy [57]. Complications directly related to the prostheses include resorption of the osteo-odonto-laminar plate (7–43%), its extrusion (1–12%), and ocular hypotony resulting from leakage at the site of keratoprosthesis implantation (3–10%) [53,57]. A strong correlation has also been demonstrated between laminar resorption and endophthalmitis caused by bacterial or fungal infections [58,59,60]. In particular, the incidence of endophthalmitis has been associated with poor oral hygiene prior to elective surgery [6,61,62,63].

Complications associated with the AlphaCor keratoprosthesis include device extrusion (8–20%), corneal stromal melting (keratolysis) (10–25%), and the formation of intraocular calcium or pigment deposits associated with the topical use of corticosteroids and β-blockers (5–15%). Although AlphaCor is considered to have a lower complication rate than other keratoprostheses, stromal melting and deposit formation remain significant limitations to achieving sustained clinical benefit [37,64,65,66].

The CorNeat keratoprosthesis appears to be associated with a relatively low complication rate; however, clinical experience with its use remains limited. Potential intraoperative complications include hemorrhage and inadequate sealing at the interface between the implant and ocular tissues. In terms of postoperative complications, conjunctival retraction (5–15%) may predispose the implanted eye to infection and increase the risk of surgical failure [38].

The EndoArt keratoprosthesis has a relatively high rate of implant dislocation and considerable variability in implantation techniques; moreover, it carries the risk of rapid detachment of the implant from the corneal stroma in a substantial proportion of eyes (partial or complete implant dislocation in approximately 15–40%) [42,67,68]. The frequency of rebubbling procedures in patients with altered anterior segment anatomy is also relatively high (20–50%) [38]. Other reported complications include elevated intraocular pressure (5–15%), ocular pain, conjunctivitis, the need for implant removal due to inadequate adherence (5–20%), subepithelial fibrosis, and keratitis [42,46,67].

Finally, glaucoma is a complication across procedures with all types of keratoprostheses (30–75%). Patients with severe, advanced ocular surface diseases who qualify for keratoprosthesis implantation, as well as those who have already undergone the procedure, frequently develop glaucoma. Multiple mechanisms may contribute to its pathogenesis: the primary disease process may involve the trabecular meshwork; the iridocorneal angle may narrow due to peripheral anterior synechiae; and conjunctival scarring and ocular surface keratinization may impair venous outflow through the episcleral veins. Additionally, prolonged corticosteroid use may contribute to both the development of de novo glaucoma and the progression of pre-existing disease. However, detecting glaucoma and assessing its progression in patients with ocular surface disorders remains challenging due to limited fundus visualization and inaccuracies in intraocular pressure measurements [18,55,69].

## 6. The Future of Prosthetic Corneal Surgery: Improved Biomaterials and Biointegration

Successful keratoprosthesis engineering depends not only on device architecture, but also on rational material selection based on specific clinical needs [70]. Both natural and synthetic polymers have been utilized as scaffolds and stromal substitutes. Natural polymers are characterized by high biocompatibility, whereas synthetic polymers enable precise control over chemical and mechanical properties. An ideal biomaterial should be biocompatible, transparent, mechanically robust, and non-immunogenic; possess appropriate refractive properties; allow permeability of oxygen and nutrients; and demonstrate resistance to neovascularization [1,16].

However, no single biomaterial fulfills all functional and biological requirements of an ideal artificial cornea. Therefore, material selection in keratoprosthesis design should be considered an indication-driven strategy in which the physicochemical properties of the material are matched to the anatomical location, mechanical demands, optical function, host tissue environment, and intended duration of implantation.

Rigid polymers such as polymethyl methacrylate (PMMA) remain particularly suitable for central optical components because of their excellent transparency, dimensional stability, and long-term optical performance. Nevertheless, their limited intrinsic bioactivity and relatively hydrophobic surface may reduce tissue integration and increase susceptibility to protein deposition or biofilm formation [71].

Hydrogels and other water-containing polymers are more attractive for peripheral interfaces and soft keratoprosthesis platforms because they provide flexibility, hydration, and improved compatibility with surrounding tissues. Their limitations include lower mechanical strength, potential swelling, and time-dependent degradation [72,73].

Porous, electrospun, and nanofibrous materials may be preferred when cellular infiltration, fibroblast anchorage, and long-term biointegration are desired. Furthermore, composite and hybrid biomaterials are increasingly being developed to combine the optical advantages of rigid polymers with the biological benefits of soft or bioactive matrices.

Another area of future research and advances is corneal endothelial tissue engineering, where the development of an optimal scaffold is considered a major objective. A functional substitute for the corneal endothelium–Descemet membrane complex should combine high optical transparency (≥90% light transmission), appropriate biomechanical compliance and elasticity (50–150 kPa), semipermeability enabling nutrient exchange and maintenance of stromal deturgescence, the ability to support adhesion and formation of a functional endothelial monolayer, and sufficient mechanical strength to withstand surgical manipulation [43,74].

The long-term success of artificial corneas depends not only on the intrinsic properties of the implanted material, but also on its ability to integrate effectively with host tissues while supporting normal cellular functions—a property referred to as biointegration. Therefore, in parallel with efforts to optimize the central implant itself, considerable attention has also been directed toward improving the interface between the biomaterial and host tissues in order to optimize biointegration. This is a dynamic biological process through which an implanted biomaterial becomes structurally and functionally incorporated into the surrounding tissue while maintaining long-term tolerance, stability, and optical performance. In corneal prosthetic surgery, successful biointegration is essential for implant retention, preservation of the visual axis, prevention of microbial ingress, and reduction in chronic inflammation, melting, or extrusion [75,76]. Clinically, insufficient biointegration may manifest as persistent epithelial defects, sterile keratolysis, conjunctival retraction, wound leakage, infection, RPM formation, stromal necrosis, or extrusion. In contrast, successful cell–material interactions improve long-term retention, reduce complication burden, enhance barrier function, and may prolong functional visual rehabilitation. Therefore, optimization of biointegration remains one of the central objectives in next-generation keratoprosthesis and corneal biomaterial design.

The process of biointegration begins immediately after implantation, with rapid adsorption of tear film proteins, extracellular matrix molecules, and inflammatory mediators onto the biomaterial surface. This provisional protein layer governs subsequent cellular recognition of the implant and is strongly influenced by surface chemistry, charge, roughness, wettability, and nanostructure [77,78]. Cell attachment is mediated primarily through integrin receptors, which interact with adsorbed proteins such as fibronectin, laminin, vitronectin, and collagen. Furthermore, activation of integrin signaling regulates cytoskeletal organization, migration, proliferation, and extracellular matrix remodeling. Depending on the anatomical location of the implant, these interactions may promote epithelial sealing, stromal fibroblast colonization, or endothelial monolayer attachment [79,80]. Surface hydrophilicity and micro-/nanotopography play major roles in modulating host response. Moderately hydrophilic surfaces generally improve epithelial coverage and reduce nonspecific protein fouling, whereas excessively hydrophobic materials may enhance bacterial adhesion and biofilm formation. Nanofibrous or porous architectures can facilitate cellular infiltration and collagen deposition, thereby improving mechanical anchorage, as demonstrated in subconjunctival integration systems such as CorNeat [81,82,83].

Macrophages are central regulators of long-term material tolerance. Predominance of a pro-inflammatory M1 phenotype may lead to fibrosis, chronic inflammation, tissue melting, or implant failure, whereas transition toward a reparative M2 phenotype is associated with constructive remodeling and stable tissue integration. Consequently, increasing attention has been directed toward immunomodulatory surfaces, heparinized coatings, peptide-functionalized scaffolds, and bioactive hydrogels capable of directing favorable host responses [84,85,86].

Recent translational strategies have focused on actively enhancing cell–material interactions to improve biointegration. For example, amphiphilic artificial endothelial layers coated with bioactive molecules such as fibronectin have been shown to increase endothelial cell adhesion by up to 50%, improve cell viability, and reduce inflammatory activation [87]. Similarly, heparin-containing coatings may decrease inflammatory marker expression by approximately 30% while enhancing stromal cell attachment in experimental models [88]. Furthermore, in vitro studies on stimuli-responsive hydrogels have also demonstrated an approximately 20% greater capacity to stimulate corneal stromal cell proliferation in comparison with conventional hydrogels [89]. Other strategies include surface modifications, the addition of drug-eluting layers, and gene-modulation approaches that can selectively silence or activate pathways involved in host inflammatory and immunological responses [70,90].

Overall, the evolution of corneal substitutes reflects a transition from passive structural implants toward biologically interactive and multifunctional materials designed to actively support tissue repair and long-term biointegration (Table 3).

### 6.1. Scaffolds

Over the past decade, research on artificial corneas has been largely driven by bioengineered scaffolds that mimic the structure and function of native corneal tissue. Scaffolds are classified by the source material: natural materials of biological origin, synthetic materials designed for specific physicochemical properties, and hybrid materials combining natural and synthetic components [91].

#### 6.1.1. Natural Scaffolds

Hydrogels are widely used natural material scaffolds due to their viscoelasticity, optical transparency, and support for cell growth and differentiation, properties that make them suitable for corneal tissue engineering [91,92]. In recent years, hydrogels have evolved into highly bioactive materials amenable to bioorthogonal chemical modifications [73]. PEG-based hydrogels exhibit very high and long-term transparency (light transmittance, 95% to 98%) and mechanical properties similar to those of native corneal tissue (elastic modulus, approximately 1.5 MPa). Hydrogels can also be functionalized with bioactive peptides to improve their biological performance: approximately 40% increases in epithelial cell adhesion and proliferation compared with non-functionalized scaffolds have been reported. However, it should be emphasized that PEG-based hydrogels exhibit limitations, including time-dependent swelling and gradual material degradation; this is a deterrent for their long-term application [93]. An alternative approach involves hyaluronic acid-based hydrogels, which exhibit an exceptional capacity for water retention, reaching approximately 1000% of their dry weight. These materials may also accelerate corneal wound healing, enabling epithelial defect closure within approximately five days following application [94]. Another limitation of PEG-based hydrogels is that they are generally softer and exhibit lower mechanical strength. Therefore, in many cases, they require additional structural reinforcement or chemical modification.

#### 6.1.2. Synthetic Scaffolds

Collagen, the principal structural protein of the corneal extracellular matrix, is commonly used in synthetic scaffolds and is a key component of many biomaterials in tissue engineering. Collagen-based scaffolds enable the reconstruction of distinct corneal layers and, thereby, provide functional properties characteristic of individual corneal structures, as well as appropriate mechanical strength and high biocompatibility. Recent advances in collagen-based bioscaffolds include the use of recombinant human collagen (RHC). Scaffolds fabricated from RHC demonstrate high clinical efficacy—approximately 85% in restoring visual function within two years following implantation—while eliciting minimal immunological responses, including inflammatory reactions observed in fewer than 5% of patients [95,96].

A bioengineered corneal implant made of RHC type III (RHCIII) has been studied for patients at high risk of graft failure. This implant demonstrated good biointegration, supported corneal epithelial regeneration, partially restored the stromal layer, and promoted nerve fiber regeneration, as evidenced by the presence of the corneal reflex [97,98]. However, RHCIII implants occasionally caused neovascularization when used in high-risk patients with inflamed or scarred corneas, and they proved most suitable for patients at low risk of rejection. To reduce the risk of neovascularization in high-risk patients, modified RHCIII implants incorporating the synthetic phospholipid methacryloyloxyethyl phosphorylcholine have been developed. After one year of follow-up, these implants were found to be free from neovascularization [99,100]. However, this observation is based on relatively short-term data, and longer follow-up is required to confirm sustained anti-angiogenic effects.

An alternative strategy for inhibiting neovascularization involves integrating nanosystems that enable sustained release of anti-vascular endothelial growth factor (VEGF) agents with acellular, biosynthetic scaffolds [101]. The literature also reports attempts to implant a synthetic cornea using anterior lamellar keratoplasty with RHCIII and 2-methacryloyloxyethyl phosphorylcholine. Preliminary findings suggest that this approach may be particularly suitable for patients with mild corneal pathologies [102], but further investigations are required to validate these findings in all patient subsets and their clinical applications. Moreover, collagen scaffolds are also prone to degradation over time, which may affect their mechanical stability. Therefore, investigations into their long-term viability are important.

#### 6.1.3. Hybrid Scaffolds

Nanotechnology has significantly advanced the design of corneal scaffolds based on hybrid materials and contributed to the development of composites with enhanced mechanical and optical properties [103]. Mixed materials incorporating glass or metallic nanoparticles exhibit greater mechanical strength and improved resistance to degradation over time, thus representing a substantial advancement in implant durability [104]. To date, nanocomposite scaffolds offer the greatest mechanical strength and the ability to precisely tailor material properties. By incorporating nanomaterials, they can achieve mechanical parameters comparable to those observed in the native cornea. For example, graphene oxide-based nanocomposites increase mechanical strength by up to 25% while preserving about 90% optical transparency for up to 12 months after implantation. Thus, the incorporation of graphene oxide appears to significantly enhance material durability and structural integrity. However, it should be emphasized that the long-term biocompatibility of these materials, as well as potential immune responses associated with their use, remains insufficiently understood and requires further investigation [105]. Moreover, the design of such materials requires strict control over composition and microstructure in order to maintain high optical transparency and appropriate biocompatibility.

### 6.2. Three-Dimensional Bioprinting

There has been considerable interest in the application of 3D bioprinting technologies in the fabrication of synthetic corneal substitutes. Three-dimensional bioprinting is a promising strategy for corneal tissue engineering, as it can enable the production of scaffolds with a layered architecture that closely mimics the natural lamellar structure of the human cornea [105,106]. This method involves layer-by-layer deposition of specialized bioinks to allow for the reproduction of the complex cellular organization and extracellular matrix of the native cornea [107]. The technology enables high-resolution printing at scales below 50 µm, thus allowing for precise replication of individual corneal layers, including the epithelium, stroma, and endothelium. As a result, bioprinting enables the fabrication of accurate, functional anatomical structures by combining appropriately selected bioinks with high-resolution deposition techniques. This technology holds potential not only for stromal reconstruction but also for generating full-thickness, biomimetic models of the entire cornea in the future [1].

The wide availability of biomaterials for corneal bioengineering enables the design of inks and bioinks that can recreate the native microenvironment of the tissue. Currently, particular attention is being paid to the reconstruction of synthetic corneal stroma using various bioprinting techniques, including inkjet, extrusion-based, and laser-assisted printing [108]. The use of diverse bioinks, such as hydrogels, collagen, and nanomaterials, can provide the combined benefits of their complementary properties, including high optical transparency, sufficient mechanical strength, and biocompatibility with native corneal tissue, while minimizing their individual limitations. Consequently, 3D bioprinting enables the precise deposition of materials with distinct properties and, thus, facilitates the fabrication of structures with well-defined biological and mechanical functions [109].

### 6.3. Other Emerging Innovations

One of the most promising directions involves the development of smart biomaterials, including stimuli-responsive hydrogels that can react to environmental changes such as pH, temperature, oxidative stress, or inflammatory mediators. These systems may enable controlled drug release, modulation of wound healing, and reduction in postoperative inflammation [73,110,111]. Nanotechnology has also introduced new possibilities in corneal tissue engineering. For example, electrospun nanofibrous scaffolds can reproduce the fibrillar architecture of the corneal extracellular matrix and enhance cellular adhesion, migration, and proliferation. Furthermore, nanoparticles are being investigated as carriers for antibiotics, corticosteroids, anti-VEGF agents, and gene-editing molecules, which can allow targeted therapy with prolonged bioavailability. In addition, nanocomposite materials containing graphene oxide, silica nanoparticles, or metallic nanostructures may improve mechanical strength, transparency, and antimicrobial performance [112,113].

Another rapidly developing area is biomimetic scaffold design based on decellularized extracellular matrix components, peptide-functionalized surfaces (e.g., Arginina, Glicyna, Dasparaginian, Arg–Gly–Asp, RGD motifs), and stem-cell compatible matrices. Combined with 3D bioprinting technologies, these approaches may enable fabrication of patient-specific corneal substitutes with customized curvature, thickness, and biomechanical properties [114,115].

Table 4 summarizes the principal differences between traditional and next-generation biomaterials used for corneal substitutes. Conventional materials such as PMMA, PHEMA, collagen, and PEG-based hydrogels provide essential structural support and optical transparency, but their biological activity and long-term tissue integration remain limited. In contrast, advanced biomaterials are designed not only to replace damaged tissue, but also to actively promote regeneration, modulate inflammation, enhance cellular interactions, and optimize biomechanical performance. Although many of these technologies remain at the preclinical stage, they represent an important step toward safer, more durable, and biologically integrated artificial corneas. Importantly, native corneal tissue remains the reference standard for all artificial substitutes.

### 6.4. Immunological Regulation

The long-term success of artificial cornea implantation depends largely on effective regulation of the host immune response. Unfortunately, immune-mediated rejection remains a principal challenge that limits the integration and durability of corneal prostheses. Specifically, pro-inflammatory cytokines, such as tumor necrosis factor alpha (TNF-α) and interleukin-6 (IL-6), play key roles in starting and sustaining inflammatory responses that lead to immune rejection, by promoting macrophage activation, T-lymphocyte infiltration, and fibrotic processes. To mitigate these adverse immune reactions, immunomodulatory strategies using modified biomaterials are currently being researched and developed. Experimental studies indicate that functionalizing artificial corneas with heparin or bioactive molecules, such as fibronectin, can markedly attenuate inflammatory responses by directing macrophage polarization toward an anti-inflammatory phenotype. In parallel, decellularized extracellular matrix scaffolds are being developed to further enhance immunological compatibility while preserving key bioactive cues necessary for tissue regeneration and integration. Decellularization techniques enable the removal of immunogenic cellular components while maintaining the structural and biochemical integrity of the extracellular matrix, which plays a crucial role in corneal regeneration processes [74,104].

### 6.5. Genetic Modifications

To reduce the risk of immune-mediated rejection of artificial corneas, researchers are also investigating innovative genetic modification strategies with promising therapeutic potential. One of the most groundbreaking tools in this field is CRISPR–Cas9 gene-editing technology, which enables precise genome modification to enhance the immunological compatibility of transplanted tissues. CRISPR-Cas9 is typically administered using specialized carriers to enable transport across the blood-eye barrier. Particular attention has been given to modulating the expression of human leukocyte antigen (HLA) class I antigens, which play a critical role in the presentation of peptides to cytotoxic T lymphocytes and constitute a key component of the mechanisms responsible for immune recognition and graft rejection. Selective silencing or elimination of specific HLA class I molecules using CRISPR–Cas9 technology may reduce recognition of transplanted tissue by the host immune system, thereby decreasing the risk of rejection [116,117]. The effectiveness of CRISPR–Cas9-based therapies for corneal applications largely depends on the use of appropriate delivery systems, such as viral vectors, liposomes, nanoparticles, and bioactive hydrogels, which enable targeted transport of genetic material to corneal cells, enhance therapeutic stability, and reduce the risk of adverse effects. These strategies are currently being intensively explored in ocular tissue engineering and regenerative medicine as promising tools for personalized treatment of corneal disorders. While the preclinical results have been promising, this approach remains in the early stages of development and requires further investigation to assess its safety and clinical efficacy [94,106,117,118].

## 7. Artificial Intelligence Applications in Keratoprosthesis, Corneal Imaging, and Future Biomaterials

Artificial intelligence (AI) is a field of computer science focused on developing systems capable of performing tasks that traditionally require human intelligence, such as pattern recognition, data analysis, decision-making, and learning from experience. In ophthalmology, these data are most commonly presented as images obtained through various diagnostic imaging modalities, which then require clinical interpretation by specialists. AI is rapidly emerging as an important tool in modern ophthalmology and may significantly influence the future development of keratoprosthesis surgery, corneal diagnostics, and regenerative medicine. Contemporary AI systems are primarily based on machine learning (ML), deep learning (DL), and artificial neural networks (ANN) [119]. Recent advances in ML and DL have enabled automated analysis of large-scale clinical and imaging datasets, supporting earlier diagnosis, improved risk stratification, and personalized therapeutic decision-making.

In corneal diseases, AI-based models have demonstrated high diagnostic performance for detecting and classifying keratoconus, infectious keratitis, dry eye disease, endothelial dysfunction, and postoperative ectasia, using data from corneal topography, tomography, anterior segment optical coherence tomography (AS-OCT), slit-lamp imaging, and in vivo confocal microscopy.

These AI models can be used to generate diagnostic maps that support clinicians in disease detection and in assessment of corneal status before and after surgical treatment. Thus, they may facilitate earlier intervention and improve monitoring of disease progression [120]. Studies have demonstrated that CNN-based models achieve very high diagnostic performance, with reported accuracies ranging from 97.6% to 99.1% in the assessment of disease severity [119,121].

In corneal transplantation, one of the key indicators of graft survival and function is endothelial cell density of the cornea. Parameters such as cell count, polymegathism, and pleomorphism, are routinely assessed using specular microscopy. In this setting, AI may serve as a valuable tool for automated endothelial image analysis, cell segmentation, and quantitative morphological assessment with high accuracy. In this way, AI models may support the early detection of graft dysfunction, postoperative monitoring, and identification of features suggestive of impending graft rejection even before overt clinical signs become apparent. Thus, the implementation of such systems may contribute to optimized postoperative care, individualized follow-up strategies, and improved long-term graft survival. However, successful clinical integration of AI requires careful external validation, model transparency, and assessment of the risk of erroneous diagnostic decisions. Particular attention should be paid to the so-called “black-box” issue, in which an algorithm generates predictions without full interpretability of the decision-making process for the clinician [119].

From a surgical perspective, AI may contribute to improved patient selection for keratoprosthesis implantation by integrating data on multiple prognostic variables, including ocular surface status, tear film function, glaucoma risk, previous graft failure, and anatomical abnormalities of the anterior segment. Predictive models may help estimate the probability of device retention, visual improvement, or postoperative complications such as RPM formation, extrusion, or elevated intraocular pressure.

AI may also play an important role in the field of corneal bioengineering. Computational models can accelerate the development of novel biomaterials by predicting optical transparency, oxygen permeability, surface wettability, mechanical strength, degradation kinetics, and cellular compatibility. Furthermore, ML-assisted material screening may reduce development time and improve the design of hybrid scaffolds intended for long-term biointegration [122]. In tissue engineering and 3D bioprinting, AI-based optimization systems may support precise control of bioink composition, print architecture, layer thickness, and cell distribution, enabling the fabrication of patient-specific corneal substitutes that more closely replicate the native corneal microstructure. Such technologies may be particularly valuable in the future development of personalized artificial corneas [123,124].

Despite these promising advances, several challenges remain, including limited availability of high-quality annotated datasets, insufficient external validation across diverse populations, lack of algorithm transparency, limits to regulatory approval pathways, and ethical considerations related to clinical implementation. Nevertheless, the integration of AI with biomaterials science and regenerative ophthalmology represents a highly promising direction that may substantially improve outcomes in patients with severe corneal blindness.

Ophthalmic surgery is among the most demanding surgical disciplines, as procedures are performed on a microscale within a confined operative field and require exceptional precision under high magnification. The development of surgical robotics may support the surgeon by reducing hand tremor, improving motion stability, enhancing the precision of instrument manipulation, and providing better control during maneuvers performed within extremely small anatomical spaces. An additional advantage of robotic systems is their potential integration with advanced imaging platforms, which may improve tissue visualization and procedural safety [125].

Beyond conventional robotic surgery and intelligent surgical instruments, increasing attention has also been directed toward cybersurgery. This term refers to remote procedures in which a surgeon operates on a patient located at a distant site using telemanipulation systems and real-time data transmission. In the future, such solutions may expand access to highly specialized ophthalmic procedures, particularly in regions with limited access to expert surgical care.

Despite these potential benefits, the implementation of robotics in ophthalmology—especially in corneal surgery—also faces important limitations. These include high acquisition and maintenance costs, restricted availability of technology, the need for dedicated training programs, longer implementation pathways, and the necessity for further studies demonstrating clear clinical advantages over conventional techniques [119].

## 8. Translational Readiness, Regulatory Challenges and Future Clinical Adoption

Although several emerging technologies in corneal bioengineering demonstrate substantial therapeutic promise, their successful transition into routine clinical practice depends not only on scientific innovation, but also on translational readiness, regulatory approval, manufacturing scalability, and long-term safety. The current maturity of established and emerging corneal technologies remains heterogeneous. Their relative translational readiness and principal barriers to adoption are summarized in Table 5 [8,42,43,94,104,106,107,126].

Overall, the translational readiness of available and emerging corneal substitutes remains heterogeneous. While established devices such as the Boston keratoprosthesis and MOOKP are currently used in specialized clinical settings and represent the highest level of translational implementation, newer platforms—including endothelial keratoprostheses, smart biomaterials, nanocomposite scaffolds, stem-cell-based constructs, and 3D-bioprinted corneal substitutes—remain at various stages of preclinical development, pilot clinical evaluation, or early translational adoption [8].

A major challenge for next-generation corneal technologies is the demonstration of long-term biocompatibility, optical stability, mechanical durability, and predictable integration with host tissues. For cell-based and gene-modified approaches, additional concerns include genomic safety, phenotypic stability, sterility, reproducibility, and storage logistics. Similarly, nanomaterial-containing platforms require comprehensive toxicological assessment, including evaluation of local inflammatory responses, biodegradation products, and potential long-term tissue accumulation [127].

Regulatory pathways also represent a critical barrier to implementation. Depending on their composition and mechanism of action, advanced corneal substitutes may be classified as medical devices, combination products, tissue-engineered constructs, or advanced therapy medicinal products, each associated with distinct approval requirements. Standardization of manufacturing processes, compliance with Good Manufacturing Practice (GMP), validated quality-control protocols, and post-market surveillance strategies will be essential for successful translation [128].

The economic and logistical considerations related to these devices must also be addressed. High production costs, the need for specialized surgical training, limited infrastructure, and restricted access to advanced manufacturing technologies may hinder broad clinical dissemination, particularly in low-resource settings where corneal blindness remains highly prevalent.

Despite these challenges, ongoing interdisciplinary collaboration between ophthalmologists, biomaterial scientists, engineers, regulatory experts, and industry partners is expected to accelerate the development of safer, more durable, and accessible artificial corneal solutions. A realistic appraisal of translational readiness is, therefore, essential when defining future priorities in the field of keratoprosthesis and corneal regeneration [43,94,129,130].

## 9. Conclusions

Keratoprostheses represent a significant advancement in the management of patients with advanced corneal blindness for whom conventional corneal transplantation is ineffective. They represent a last-resort intervention for corneal blindness, particularly in patients with end-stage ocular surface disease and in those at high risk of failure of conventional penetrating keratoplasty. Keratoprosthesis procedures require specialized preparation of the implantation devices and a high level of commitment to long-term follow-up and postoperative care. The selection of appropriate keratoprosthesis based on a patients’ indications is a critical determinant of treatment success.

The first part of this review presented the current knowledge on available keratoprostheses, their properties, indications for their use, and associated complications. Keratoprostheses can mainly be classified as rigid and soft types based on the material used. The former offers better mechanical strength and long-term stability, while the latter offers better biointegration. Each type has its strengths and limitations, and treatment decisions need to be made according to patient indications, risk assessment, and resource availability in the clinical setting.

The second part of this review delved into current research on innovative materials and technologies to create an “ideal” keratoprosthesis—one that incorporates an optimal biomaterial and exhibits optimized biointegration capabilities. Rapid advances in biomaterials science and 3D printing may, in the future, enable the fabrication of fully functional biosynthetic corneas with programmable spatial, optical, and biomechanical properties that are not achievable with conventional corneal transplantation [108]. Early results from studies on bioengineered scaffolds and synthetic corneal substitutes indicate the potential of innovative biomaterials to restore both the structure and function of the cornea with minimal immunological risk. Approaches to improve biointegration include the functionalization of artificial corneas with bioactive molecules (e.g., heparin) that can attenuate inflammatory reactions, decellularization of the extracellular matrix to eliminate immunogenic cellular components, and genetic modulation of antigens that trigger host immune reactions and rejection [74,88,104,116,117,131,132,133].

While the results of the literature presented here are promising, it must be noted that a large part of the recent research on new biomaterials and their optimization is in the preclinical stages and needs further research before the findings can be translated and applied in the clinical setting. Further development of these advanced biomimetic materials and implantation techniques could pave the way for more effective, less invasive treatment protocols and improved quality of life for patients with severe corneal diseases.

## 10. Limitations

Despite significant advances in keratoprostheses design, their clinical application remains limited by several complications and challenges. These include the risk of infection, glaucoma progression, RPM formation, and long-term structural instability. Furthermore, most keratoprostheses require lifetime follow-up, intensive postoperative care, and, in many cases, multiple interventions. Importantly, visual outcomes can be limited by coexisting ophthalmologic conditions, particularly those affecting the retina and optic nerve. Furthermore, patient selection remains crucial, as treatment outcomes depend heavily on ocular surface health and the underlying disease. Overall, although keratoprostheses represent a valuable option for restoring vision in otherwise incurable cases, their use remains limited by a significant complication rate, the need for lifetime treatment, and limitations related to both device design and patient-specific factors.

Research regarding the innovations in biomaterials, biointegration capabilities, and AI applications is still in its early stages and currently carries limited translational relevance.

## 11. Future Directions

Future advances in keratoprostheses design should focus on improving long-term biointegration, reducing complication rates, and improving functional visual outcomes. Advances in biomaterials, particularly bioengineering and hybrid scaffolds, may enable better mimicking of the natural properties of the cornea while maintaining sufficient mechanical stability. Furthermore, the introduction of anti-inflammatory, anti-fibrotic, and anti-angiogenic agents may help reduce complications, including neovascularization and RPM formation, thereby improving long-term implant survival. Emerging technologies, including 3D bioprinting, regenerative methods, and AI-assisted prediction and analysis, may enable the development of patient-specific implants with improved structural and optical properties, as well as improve diagnosis and treatment decisions.

Future research in this field needs to focus on creating a better understanding of implant–host interactions and the immune response, both of which are crucial to optimizing long-term outcomes. Furthermore, robust clinical trials with extended follow-up will be necessary to better evaluate the durability, safety, and functional effectiveness of newly developed keratoprostheses, especially in high-risk patient populations. Importantly, the future value of emerging keratoprosthesis technologies will depend not only on technical feasibility, but also on whether they can meaningfully improve safety, long-term durability, accessibility, and patient-centered visual outcomes compared with current standards of care. Future progress will, therefore, require not only material innovation, but also robust clinical validation, scalable manufacturing pathways, and equitable global access to advanced corneal care.

## Figures and Tables

**Table 1:** Integrated Comparative Analysis of Major Keratoprosthesis Platforms.

Parameter	Boston Keratoprosthesis (Type I/II)	OOKP/MOOKP	CorNeat Keratoprosthesis	EndoArt
Primary indication	End-stage corneal blindness with relatively preserved ocular surface (Type I); severe cicatrizing ocular surface disease (Type II)	Bilateral corneal blindness with severe dry eye, keratinization, lid dysfunction, and end-stage ocular surface failure	Advanced corneal blindness unsuitable for conventional grafting; donor-independent alternative in selected high-risk eyes	Chronic corneal edema due to endothelial dysfunction, especially in eyes at high risk of graft failure
Target corneal layer	Full-thickness optical replacement	Full-thickness optical replacement	Full-thickness optical replacement	Endothelial replacement/posterior corneal barrier
Need for donor tissue	Yes (carrier cornea required)	No donor cornea; uses autologous tooth/bone and mucosa	No donor cornea required	No donor endothelium required
Main fixation principle	Donor carrier graft secured between front and back plates	Autologous osteo-dental lamina with optical cylinder	Subconjunctival nanofiber skirt integration	Posterior stromal apposition with gas tamponade ± fixation sutures
Main materials	PMMA ± titanium + donor cornea	PMMA optical cylinder + autologous biological tissues	Synthetic polymer nanofiber scaffold + optical core	Hydrophilic acrylic copolymer
Biointegration potential	Moderate; dependent on carrier graft and ocular surface health	Very high; durable autologous tissue integration	Potentially high; fibrovascular subconjunctival integration	Limited; mainly mechanical adherence to posterior stroma
Surgical complexity	Moderate (Type I) to high (Type II)	Very high; multistage and multidisciplinary surgery	Moderate; less invasive than OOKP	Moderate; lamellar intraocular procedure
Visual rehabilitation potential	Good to very good in selected patients	Good functional vision in appropriately selected severe cases	Promising, but long-term data limited	Improvement through edema resolution rather than optical cylinder implantation
Major complications	Glaucoma, retroprosthetic membrane formation, keratolysis, infection, extrusion	Glaucoma, lamina resorption, mucosal complications, retinal complications, endophthalmitis	Limited data; conjunctival retraction, wound leakage, infection risk, implant failure	Detachment/dislocation, rebubbling, fibrosis, elevated IOP, explantation
Reported retention/survival	Approx. 75–90% at 5 years	Approx. 70–90% long-term (selected expert centers)	Early short-term success reported	Good attachment in most reported cases after repositioning if needed
Long-term evidence	Extensive	Extensive in expert centers	Early clinical evidence	Early to intermediate clinical evidence
Best suited patient profile	Multiple graft failures with usable ocular surface anatomy	Most severe forms of ocular surface disease with absent tears/blinking	Patients requiring synthetic donor-independent alternative	Endothelial decompensation with high transplant risk
Translational maturity	High	High	Moderate	Moderate
Main limitation	Lifelong surveillance and glaucoma burden	High surgical complexity and limited availability	Limited long-term outcomes	Narrow indication spectrum and evolving technique

Abbreviations: OOKP, osteo-odonto-keratoprosthesis; MOOKP, modified osteo-odonto-keratoprosthesis; PMMA, polymethyl methacrylate; IOP, intraocular pressure.

**Table 2:** Quantitative Outcomes and Clinical Evidence for Major Keratoprosthesis Platforms.

Parameter	Boston Keratoprosthesis	OOKP/MOOKP	CorNeat Keratoprosthesis	EndoArt
Reported anatomical retention/device survival	Approx. 75–90% at 5 years in major series	Approx. 70–90% long-term retention in specialized centers	Early short-term retention reported in first-in-human and preclinical studies	High attachment rates reported after repositioning when required
Visual acuity improvement	Frequent and clinically significant in appropriately selected eyes	Functional visual rehabilitation in many end-stage ocular surface cases	Early restoration of transparent optical axis reported	Improvement associated with corneal deturgescence and edema reduction
Reduction in corneal thickness/edema	Not primary endpoint	Not primary endpoint	Not primary endpoint	Significant reduction reported (e.g., >800 µm to ~550–600 µm in published series)
Glaucoma/IOP burden	High	Very high	Insufficient long-term data	Reported in selected series, especially complex eyes
Retroprosthetic membrane formation/fibrosis	Common	Less typical than Boston keratoprosthesis; other interface complications predominate	Unknown/insufficient data	Subepithelial fibrosis reported
Need for secondary procedures	Common (membranectomy, glaucoma surgery, revisions)	Common (revisions, vitreoretinal procedures, glaucoma surgery)	Unknown	Common (rebubbling, repositioning, fixation adjustments)
≥5-year follow-up available	Yes	Yes	No	No
Largest evidence base	Large multicenter and single-center clinical cohorts	Long-term expert-center case series	Early feasibility studies and small cohorts	Early clinical series and systematic review data
Evidence level	High	High	Low to moderate	Moderate

Abbreviations: OOKP, osteo-odonto-keratoprosthesis; MOOKP, modified osteo-odonto-keratoprosthesis; IOP, intraocular pressure.

**Table 3:** Traditional versus Next-Generation Biomaterials for Corneal Substitutes.

Feature	Traditional Biomaterials	Next-Generation Biomaterials
Main concept	Structural replacement of damaged tissue	Bioactive support for regeneration and long-term integration
Typical materials	PMMA, PHEMA, collagen, PEG-based hydrogels	Smart hydrogels, nanocomposites, peptide-functionalized scaffolds, decellularized extracellular matrix, bioinks
Biological activity	Limited or passive	High; supports cell adhesion, migration, proliferation, and tissue remodeling
Drug delivery capability	Usually absent	Can incorporate controlled-release therapeutic systems
Mechanical properties	Fixed, material-dependent	Tunable, programmable, and customizable
Optical transparency	Good to excellent	Designed to mimic native corneal optics while preserving transparency
Resistance to infection	Limited intrinsic protection	Can be enhanced using antimicrobial coatings, nanoparticles, or anti-biofilm surfaces
Immunological profile	Variable; may induce chronic foreign-body response depending on material	Improved through surface engineering, biomimetic chemistry, and immunomodulatory functionalization
Personalization potential	Low	High; patient-specific geometry and 3D bioprinting possible
Manufacturing strategy	Conventional industrial fabrication	Advanced manufacturing, additive manufacturing, and biofabrication
Current stage of development	Established clinical use	Preclinical to early translational development

Abbreviations: PMMA, polymethyl methacrylate; PHEMA, poly(2-hydroxyethyl methacrylate); PEG, polyethylene glycol.

**Table 4:** Mechanical and Optical Properties of Selected Biomaterials for Corneal Substitutes.

Property/Material	Native Cornea	PEG Hydrogels	Collagen-Based Scaffolds	Nanocomposite Scaffolds
Light transmission (%)	90–98	90–98	85–95	85–95
Elastic modulus/mechanical range	Approx. 0.3–1.5 MPa	Lower than the native cornea; tunable by crosslinking	Moderate; depends on density and crosslinking	High; may approach or exceed that of the native cornea
Biocompatibility	Excellent	Very high	Very high	Moderate to high (composition-dependent)
Cell adhesion potential	Native physiological baseline	Usually requires functionalization	Excellent intrinsic bioactivity	Good after surface modification
Integration with host tissue	Native tissue	Limited without surface engineering	Very good	Good after biofunctionalization
Oxygen/nutrient permeability	High	High	Relatively high	Variable; design-dependent
Mechanical stability	Excellent	Moderate	Moderate	High
Main advantages	Benchmark for transparency, biomechanics, and biological function	High transparency, flexible chemistry, drug-loading potential	Strong biomimicry, regenerative potential, excellent cell compatibility	Superior strength, tunable multifunctionality, antimicrobial potential
Main limitations	—	Swelling, gradual degradation, lower strength	Enzymatic degradation, hydration-related instability	Early-stage evidence, toxicity concerns, complex manufacturing

Abbreviations: PEG, polyethylene glycol; MPa, megapascal.

**Table 5:** Translational Readiness of Current and Emerging Corneal Technologies.

Technology	Current Development Stage	Translational Readiness	Main Barriers to Clinical Adoption
Boston Keratoprosthesis	Established clinical use	High	Long-term complications, glaucoma monitoring, need for lifelong follow-up
OOKP/MOOKP	Established in specialized centers	High	Surgical complexity, multidisciplinary expertise, limited availability
CorNeat	Early clinical implementation	Moderate	Limited long-term data, need for multicenter validation, complication profile still evolving
EndoArt	Early clinical implementation	Moderate	Limited long-term data, implant positioning, rebubbling, patient selection
Smart biomaterials/responsive hydrogels	Preclinical/early translational	Low–Moderate	Long-term stability, reproducibility, regulatory validation
Nanocomposite corneal scaffolds	Preclinical	Low	Biocompatibility, toxicity assessment, manufacturing standardization
3D-bioprinted corneal substitutes	Experimental/preclinical	Low	Scale-up production, structural durability, regulatory approval
Stem-cell-seeded corneal constructs	Early translational/pilot studies	Moderate	Cell sourcing, cost, standardization, storage and transport
CRISPR-based immunomodulation	Conceptual/preclinical	Low	Safety, off-target effects, ethical and regulatory concerns

## Data Availability

No new datasets were collected or analyzed as part of this study.

## Abbreviations

The following abbreviations are used in this manuscript:

RPM	retroprosthetic membrane
PPMA	polymethyl methacrylate
OOKP	osteo-odonto-keratoprosthesis
MOOKP	modified osteo-odonto-keratoprosthesis
LVPKP	LV Prasad Eye Institute Keratoprosthesis
PHEMA	poly(2-hydroxyethyl methacrylate)
PEG	polyethylene glycol
RHC	recombinant human collagen
RHCIII	recombinant human collagen type III
